# Association between radiomics features of DCE-MRI and CD8^+^ and CD4^+^ TILs in advanced gastric cancer

**DOI:** 10.3389/pore.2023.1611001

**Published:** 2023-06-05

**Authors:** Huizhen Huang, Zhiheng Li, Yue Xia, Zhenhua Zhao, Dandan Wang, Hongyan Jin, Fang Liu, Ye Yang, Liyijing Shen, Zengxin Lu

**Affiliations:** ^1^ Shaoxing of Medicine, Shaoxing University, Shaoxing, China; ^2^ Department of Radiology, Anhui Provincial Hospital, Hefei, China; ^3^ Department of Radiology, Shaoxing People’s Hospital, Shaoxing, China; ^4^ Country Department of Pathology, Shaoxing People’s Hospital, Shaoxing, China; ^5^ The First Affiliated Hospital of Shaoxing University, Shaoxing, China

**Keywords:** radiomics, CD4^+^, CD8^+^, advanced gastric cancer (AGC), dynamic contrast-enhanced magnetic resonance imaging (DCE-MRI)

## Abstract

**Objective:** The aim of this investigation was to explore the correlation between the levels of tumor-infiltrating CD8^+^ and CD4^+^ T cells and the quantitative pharmacokinetic parameters of dynamic contrast-enhanced magnetic resonance imaging (DCE-MRI) in patients with advanced gastric cancer.

**Methods:** We retrospectively analyzed the data of 103 patients with histopathologically confirmed advanced gastric cancer (AGC). Three pharmacokinetic parameters, K_ep_, K^trans^, and V_e_, and their radiomics characteristics were obtained by Omni Kinetics software. Immunohistochemical staining was used to determine CD4^+^ and CD8^+^ TILs. Statistical analysis was subsequently performed to assess the correlation between radiomics characteristics and CD4^+^ and CD8^+^ TIL density.

**Results:** All patients included in this study were finally divided into either a CD8^+^ TILs low-density group (*n* = 51) (CD8^+^ TILs < 138) or a high-density group (*n* = 52) (CD8^+^ TILs ≥ 138), and a CD4^+^ TILs low-density group (*n* = 51) (CD4^+^ TILs < 87) or a high-density group (*n* = 52) (CD4^+^ TILs ≥ 87). ClusterShade and Skewness based on K_ep_ and Skewness based on K^trans^ both showed moderate negative correlation with CD8^+^ TIL levels (*r* = 0.630–0.349, *p* < 0.001), with ClusterShade based on K_ep_ having the highest negative correlation (*r* = −0.630, *p* < 0.001). Inertia-based K_ep_ showed a moderate positive correlation with the CD4^+^ TIL level (*r* = 0.549, *p* < 0.001), and the Correlation based on K_ep_ showed a moderate negative correlation with the CD4^+^ TIL level, which also had the highest correlation coefficient (*r* = −0.616, *p* < 0.001). The diagnostic efficacy of the above features was assessed by ROC curves. For CD8^+^ TILs, ClusterShade of K_ep_ had the highest mean area under the curve (AUC) (0.863). For CD4^+^ TILs, the Correlation of K_ep_ had the highest mean AUC (0.856).

**Conclusion:** The radiomics features of DCE-MRI are associated with the expression of tumor-infiltrating CD8^+^ and CD4^+^ T cells in AGC, which have the potential to noninvasively evaluate the expression of CD8^+^ and CD4^+^ TILs in AGC patients.

## Introduction

Although the incidence and mortality of gastric cancer have declined over the past decades, gastric cancer remains the fourth most prevalent cancer worldwide [1]. Traditional surgical resection remains the main treatment for gastric cancer [2]. However, most patients with gastric cancer are usually diagnosed at an advanced stage, and thus less than 30% are suitable for radical resection [3]. In recent years, there have been new research advances in the treatment of advanced gastric cancer (AGC), and immunotherapy based on blocking immune checkpoint inhibitors (ICIs) has made a great promise for gastric cancer patients [4]. While immune checkpoint blockade significantly extends the survival of patients with AGC, only a small percentage of patients can benefit from treatment, in part because tumor-immune interactions in the tumor immune microenvironment (TIME) suppress the efficacy of ICI [5]. Furthermore, cell adoptive therapy based on tumor-infiltrating lymphocytes has shown promising results in the treatment of advanced solid tumors [6]. This is an immunotherapy that eliminates tumor cells by using immune cells in the tumor microenvironment.

Tumor-infiltrating lymphocytes (TILs) are an important part of the TME and are related to the metabolism of tumor cells and the local immune response. The number of T lymphocyte subsets is considered to be not only closely related to the metastasis, progression, and growth of malignant tumors, but also related to immune dysfunction, a key factor affecting the effect of tumor immunotherapy [7–9]. T lymphocytes are mainly divided into different functional subsets: CD4^+^ T cells, effector (CD8^+^) T cells, and other types of cells such as natural killer T (NKT) cells. CD8 is a marker of cytotoxic T cells and is the main antitumor effector cell, as it can recognize and kill tumor cells. The majority of CD4^+^ T cells are helper T lymphocytes. CD4^+^ T cells that promote their activation and proliferation and their synergistic antitumor activity are considered to be essential for tumor surveillance. Studies have shown that monitoring the changes in T lymphocytes in tumor patients is of great significance for the timely reflection of immune function and the prognosis of therapeutic effects in tumor patients. In addition, CD4^+^ and CD8^+^ T cells are closely associated with an improved response to immunotherapy in patients with advanced gastric cancer [10, 11]. Immune checkpoint inhibitor (ICI) therapy aims to prevent tumor cells from suppressing the immune system, thus enabling the immune system to attack tumor cells more effectively. However, the effectiveness of ICI therapy may be diminished if a patient has a low number of CD4^+^ and CD8^+^ T cells, as these cells are responsible for recognizing and killing cancer cells. Consequently, the number of CD4^+^ and CD8^+^ T cells may impact the efficacy of ICI therapy [12]. The gold standard for TIL quantification is the evaluation of biopsy specimens by immunohistochemistry. However, this approach is limited by its invasiveness, no reproducibility, and inability to reflect intra- and intertumoral heterogeneity. These shortcomings indicate an urgent need to comprehensively assess TIL levels in a dynamic and noninvasive biomarker manner.

Radiomics is an emerging field in which subtle information contained in medical imaging is extracted and transformed into quantitative features that represent the underlying tissue attributes of a tumor. Radiomics has the potential to improve our understanding of tumor biology and guide the choice of clinical treatment options [4, 13, 14]. Several studies have confirmed that radiomics is very effective for the diagnosis and classification of gastric cancer as well as the identification of other molecules and is potentially a biomarker to assess immunohistochemical features, thereby helping to guide the treatment of patients [15–17]. Several studies have discussed the correspondence between DCE-MRI and EGFR and VEGF expression levels; however, few studies have discussed radiomics assessment at the TIL level based on preoperative DCE-MRI in patients with advanced gastric cancer. Therefore, this study aims to preliminarily explore the correlation between the radiomics features of DCE-MRI and the infiltration degree of CD8^+^ and CD4^+^ tumor-infiltrating T lymphocytes and lay the foundation for future research.

## Materials and methods

### Patient demographics

Informed consent did not need to be obtained by patients in this retrospective study after the Ethics Committee of Shaoxing People’s Hospital approved it. We enrolled patients with advanced gastric cancer admitted to Shaoxing People’s Hospital between April 2018 and July 2022, and we collected their imaging data before treatment. The following criteria were used for inclusion: 1) surgery or gastroscopic biopsy confirmed GC; 2) there were no absolute contraindications to MRI; and 3) prior to DCE-MRI, no antitumor therapy was administered. The following criteria were used for exclusion: 1) heavy image artifacts, which seriously affected the delineation of the lesion; 2) maximum tumor diameter <1 cm; and 3) contraindications to surgery or puncture. According to the study’s findings, 103 patients participated, 77 males and 26 females, with an average age of 67.7 years (range 33–88 years).

### DCE-MRI examination

All patients were prepared as follows: 1) patients fasted for 8 h before examination and underwent respiratory training 30 min before the examination; 2) intramuscular anisodamine injection (Hangzhou Minsheng Pharmaceutical Co., Ltd., China) was injected 10 min before the examination to prevent gastrointestinal motility; and 3) 5 min before the MRI, patients drank 800–1,000 mL of tepid water to fill their stomachs.

A 3.0 T magnetic resonance scanner (Verio, Siemens, Germany) with a 12-channel phased-array surface coil was used for scanning. The patient was evaluated while lying flat, and the entire stomach was scanned. T1WI, T2WI, and DCE-MRI were all part of the scan’s overall sequence, and DCE-MRI was carried out while the patient was breathing freely. Initially, multi-flip angle cross-sectional T1WI was performed, and a fast 3D volume interpolation gas fat-suppression sequence was used. These were the precise specifications: slice thickness is 5 mm, the field of view is 350 mm by 284 mm, the matrix is 288 × 164, and the repetition time is 3.25 ms. Different flip angles (5°, 10°, 15°) were used to scan one stage, each cycle was 6.5 s, and the total time was 19.5 s. The gradient echo volume interpolation fat-suppression sequence was used. The flip angle was 10°, a total of 35 phases were scanned, and the imaging time was 227.5 s. The other variables were the same as for the cross-sectional T1WI with several flips. In the third phase, gadodiamide (Omniscan, GE Healthcare, China) was given at a dose of 0.1 mmol/kg and an injection rate of 3.5 mL/s. Normal saline was then flushed with 20 mL at the same flow rate.

### Image information evaluation

**FIGURE 1:**
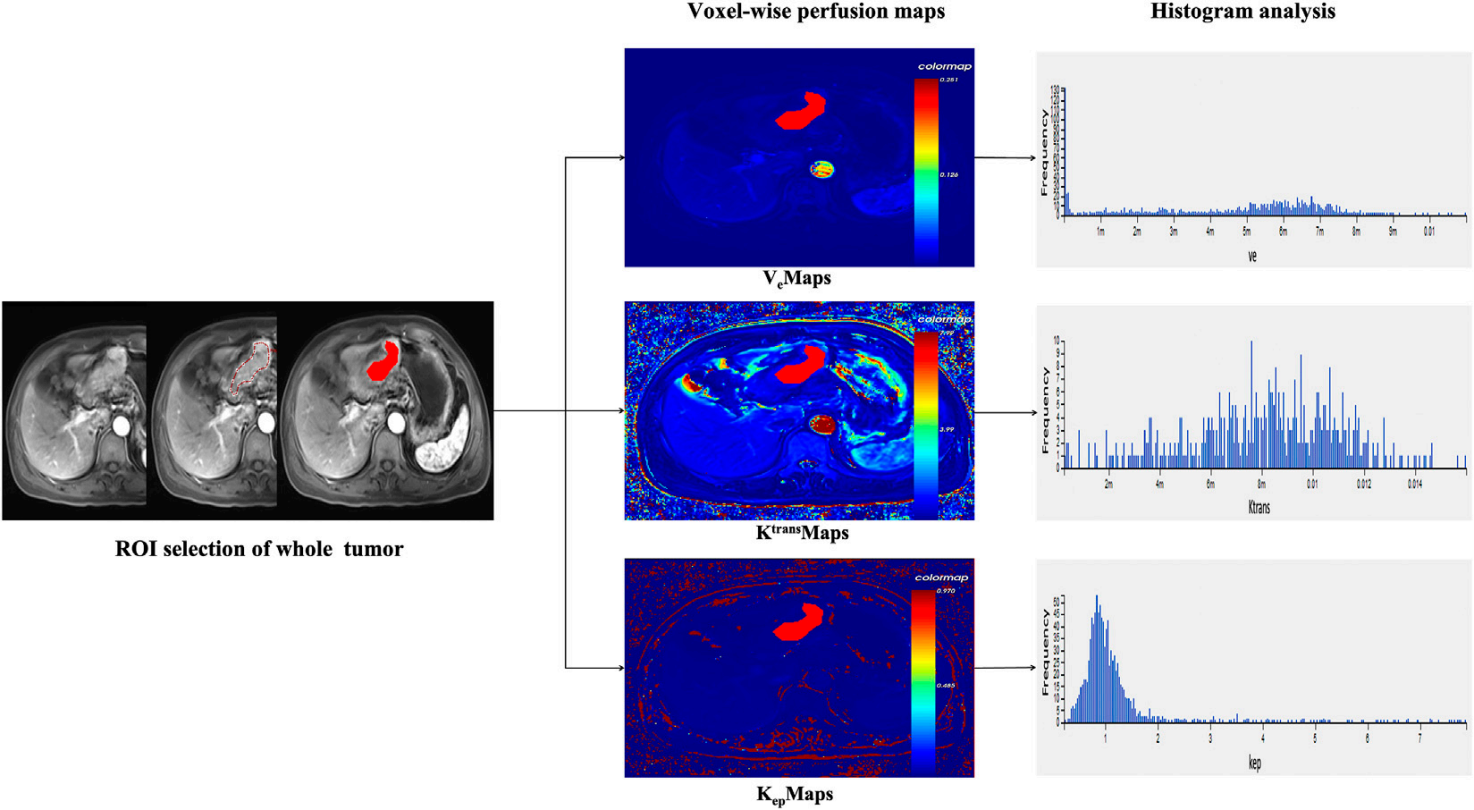
Voxel-wise analysis of the entire tumor is shown in the research’s overall flow chart.

The raw images of all eligible AGC patients were imported into the hemodynamic software Omni. Kinetics (GE Healthcare, China). Nonrigid 3D registration was used to preprocess the DCE images and Omni. Kinetics software was used to post-process the multi-flip angle (5°, 10°, 15°) and correct the dynamically enhanced images. The abdominal aorta was chosen as the entry artery. The perfusion parameters were calculated using the Tofts pharmacokinetic model (Ktrans, Kep, and Ve). The study area was chosen to avoid cystic degeneration, necrosis, and normal gastric wall tissue. Two senior radiologists delineated the contours by hand. The AGC lesion was delineated layer by layer and then fused to form a 3D ROI. From the three perfusion maps, the software generated 231 radiomics features, including Haralick, first-order, histogram, gray-level cooccurrence matrix, and run-length matrix. The final measurements were all the mean values obtained by repeating three calculations. In Figure 1, the workflow is displayed.

### Immunohistochemical staining and evaluation

**FIGURE 2:**
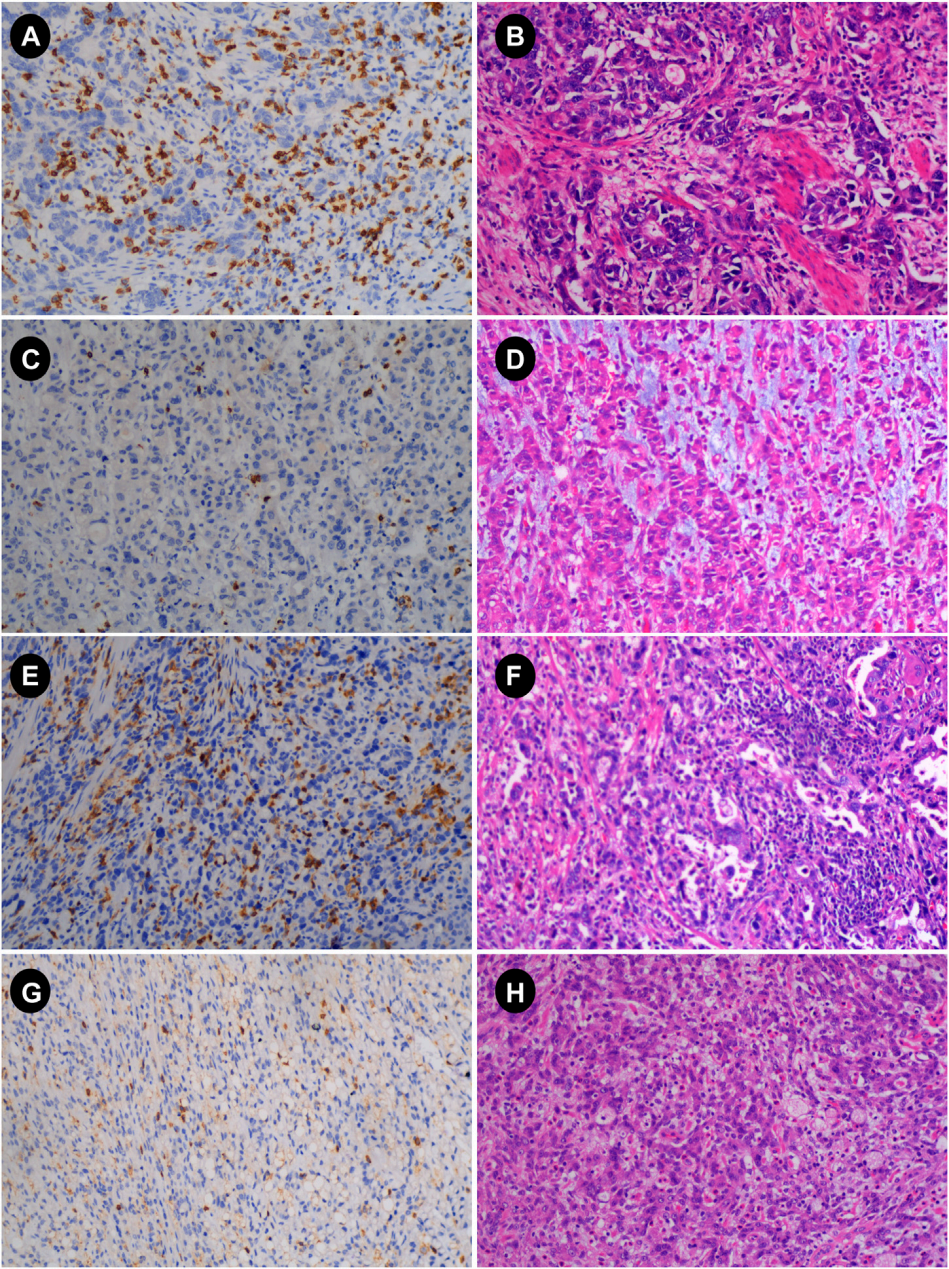
Immunohistochemistry (IHC) and hematoxylin-eosin (HE) images of advanced gastric cancer. **(A,B)** IHC and HE plots with high CD8; **(C,D)** IHC and HE plots with low CD8; **(E,F)** IHC and HE plots with high CD4; **(G,H)** IHC and HE plots with low CD4 (magnification: 10 × 40) HE, hematoxylin and eosin staining; IHC, immunohistochemistry.

The main immunohistochemistry (IHC) staining and quantitative analysis of CD4^+^ and CD8^+^ T cells was carried out in accordance with previous research [10, 18]. Gastric cancer specimens were obtained by surgery or gastroscopic biopsy. All gastric cancer specimens were cut into 4-μm-thick slices after paraffin embedding. All slices were deparaffinized and hydrated before antigen retrieval. Endogenous peroxidase activity was then inhibited in a 3% H_2_O_2_ solution for 10 min at 37°C (H36021693, Nanchang Baiyun Pharmaceutical Co., Ltd., Nanchang, China). Sections were immunohistochemically stained with rabbit anti-CD4 monoclonal antibody (GT219102, Gene Tech, Shanghai, China) or mouse anti-CD8 monoclonal antibody (GT211202, Gene Tech, Shanghai, China), and stored in the refrigerator at 4°C overnight. Sections were subsequently stained with secondary antibodies (K5009, Dako, Beijing, China) and then incubated at 37°C for 10 min. Diaminobenzidine (DAB) staining, hematoxylin counterstaining. The slides were examined under a microscope after dehydration, transparency, and mounting. The immunohistochemical results were reviewed by two experienced pathologists in a double-blind manner. The whole field of tissue was observed under a low-power microscope, and then five fields were randomly selected under high-power fields (X40) in each case (Figure 2). The counting field of view included cancer cell nests within the tumor tissue and the surrounding stroma. Then, according to previous studies [18], the expression of CD4^+^ and CD8^+^ TIL was evaluated according to the average number of positively stained cells, and then patients were divided into high and low groups according to the median.

### Statistical analyses

The data analysis and image drawing processes used SPSS (version 24, IBM Corporation, United States) and GraphPad Prism 8.0 (GraphPad Prism Software Inc., San Diego, CA, United States). The Mann‒Whitney U test or independent sample Student’s t-test was applied depending on the circumstances. Measurement data are expressed as the mean and standard deviation. When appropriate, Fisher’s exact probability method or the chi-square test was employed to describe count data as frequency (%). Due to the nonnormal distribution of continuous variables, Spearman correlation was employed to examine the relationship between each DCE-MRI perfusion parameter and the degree of CD4^+^ and CD8^+^ TIL infiltration. For all statistics, two-sided probability tests were employed. Statistics were judged significant at *p* < 0.05.

## Results

The study included 103 patients with advanced gastric cancer who presented to Shaoxing People’s Hospital between the ages of 33 and 88. The median value of CD4^+^ and CD8^+^ TILs was 87 and 138, respectively. As shown in Table 1, the study results revealed no significant differences in gender, age, BMI, tumor location, differentiation level, CEA level, CA199 level, and CA125 level between cohorts with high and low infiltration levels of CD4 and CD8.

**TABLE 1:** Advanced gastric cancer patients’ clinical features.

Characteristic	CD8^+^ TILs status			CD4^+^ TILs status		
	High (*n* = 52)	Low (*n* = 51)	P	High (*n* = 52)	Low (*n* = 51)	P
Age (years, mean ± SD)	67.673 ± 11.206	69.863 ± 9.210	0.286	68.096 ± 10.417	69.431 ± 10.185	0.516
BMI (Kg/m^2^, mean ± SD)	22.965 ± 2.887	22.383 ± 3.314	0.348	22.148 ± 2.619	23.215 ± 3.476	0.084
Gender			0.954			0.609
Male	39	38		40	37	
Female	13	13		12	14	
Location			0.211			0.091
Cardia	11	6		8	9	
Body	13	20		12	21	
Antrum	28	25		32	21	
Differentiation level			0.494			0.280
High/Moderate	30	26		31	25	
Poor	22	25		21	26	
CEA level (ng/mL)			0.945			0.442
Normal	35	34		33	36	
Elevated	17	17		19	15	
CA199 level (μ/mL)			0.238			0.777
Normal	41	35		39	37	
Elevated	11	16		13	14	
CA125 level (μ/mL)			0.446			0.854
Normal	42	38		40	40	
Elevated	10	13		12	11	

**FIGURE 3:**
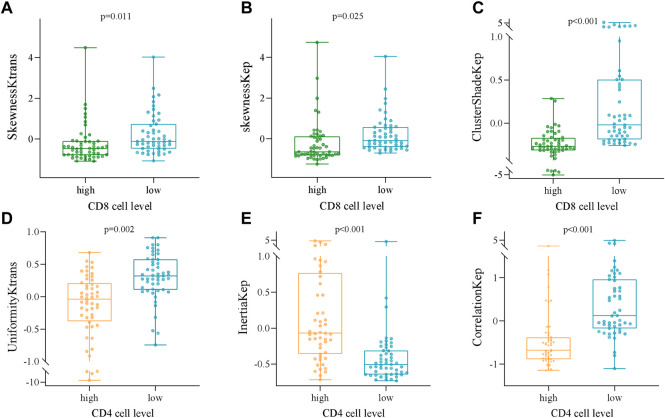
Box and whisker plots showing the relationships between the expression of CD8 and CD4 TILs and the DCE-MRI-based phenotypes. **(A)** SkewnessKtrans, **(B)** skewnessKep, **(C)** ClusterShadeKep, **(D)** UniformityKtrans, **(E)** InertiaKep, and **(F)** CorrelationKep.

Several radiomics profiles of DCE-MRI pharmacokinetic parameters significantly associated with the infiltration of CD4^+^ and CD8^+^ TILs are summarized in Figure 3 and Tables 2, 3. ClusterShade of Kep had the greatest correlation coefficient with CD8 (*r* = −0.630, *p* < 0.001), and the Correlation of Kep had the highest correlation value with CD4 (*r* = −0.616, *p* < 0.001).

**TABLE 2:** Correlation analysis between DCE-MRI parameters and CD8^+^ TILs in patients with advanced gastric cancer.

Kinetic parameter	Parameter	*r* value	*p*-value
Ktrans	Skewness	−0.349	<0.001
Kep	Skewness	−0.411	<0.001
	ClusterShade	−0.630	<0.001

Note: *r*, correlation coefficient.

**TABLE 3:** Correlation analysis between DCE-MRI parameters and CD4^+^ TILs in patients with advanced gastric cancer.

Kinetic parameter	Parameter	*r* value	*p*-value
Ktrans	Uniformity	−0.481	<0.001
Kep	Inertia	0.549	<0.001
	Correlation	−0.616	<0.001

Note: *r*, correlation coefficient.

**FIGURE 4:**
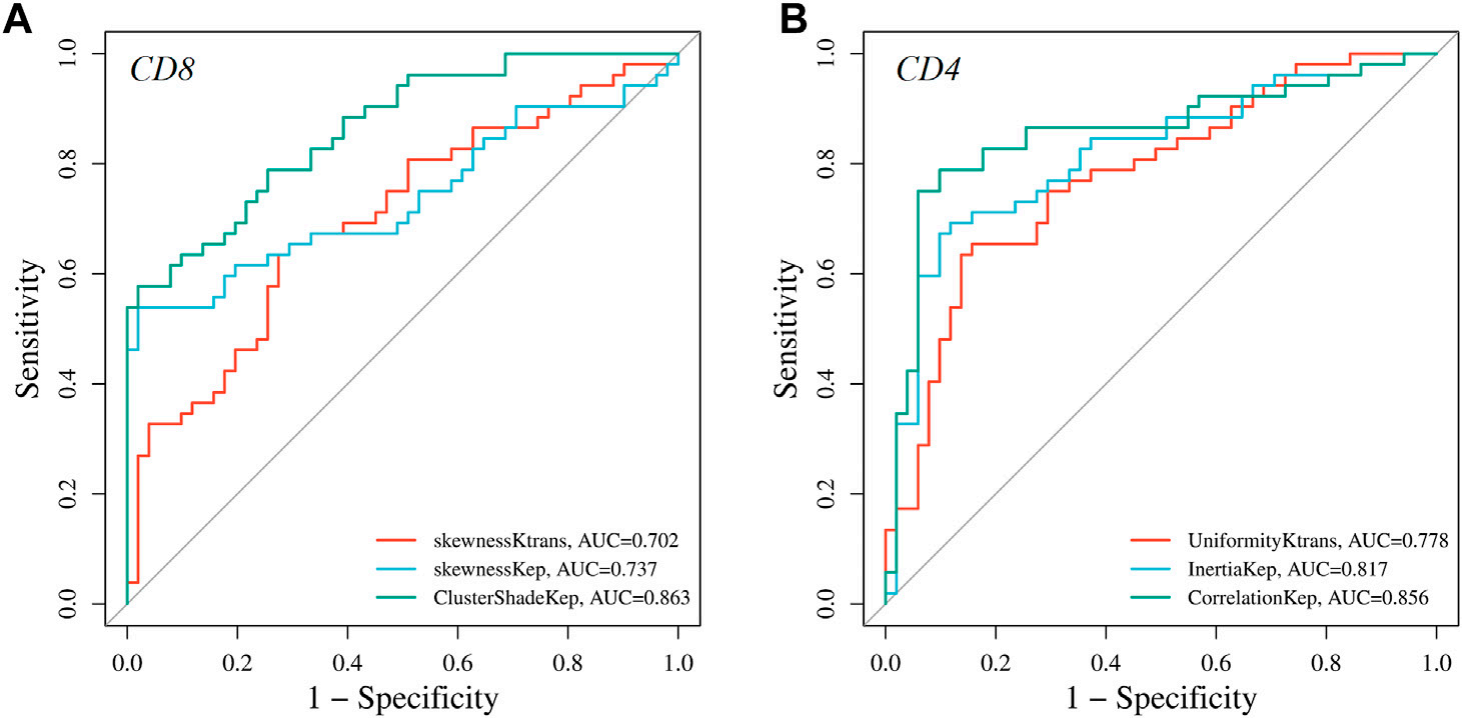
Graphs showing the receiver operating characteristic curves (ROC) of DCE-MRI parameters for differentiating high and low **(A)** CD8 and **(B)** CD4 expressions.

ROC curve analysis was performed to evaluate the diagnostic performance of radiomics parameters in distinguishing high and low expression of CD8 and CD4 in AGC. For CD8 expression, the radiomics parameters SkewnessKtrans, SkewnessKep, and ClusterShadeKep exhibited good discriminatory ability with AUC values of 0.702, 0.737, and 0.863, respectively. These parameters showed promising sensitivity, specificity, accuracy, and predictive values (Table 4 and Figure 4). Similarly, for CD4 expression, the radiomics parameters UniformityKtrans, InertiaKep, and CorrelationKep demonstrated effective discrimination with AUC values of 0.778, 0.817, and 0.856, respectively. These parameters achieved high sensitivity, specificity, accuracy, and predictive values (Table 5 and Figure 4). Overall, the results of the ROC curve analysis highlight the potential of these radiomics parameters in differentiating between high and low expression of CD8 and CD4 in AGC patients.

**TABLE 4:** ROC curve analysis of radiomics parameters for differentiating CD8 high expression from low expression in AGC.

Parameter	Cut-off	Sensitivity	Specificity	Accuracy	PPV	NPV	AUC	P
SkewnessKtrans	0.360	0.725	0.615	0.670	0.649	0.696	0.702	<0.001
SkewnessKep	0.518	0.980	0.519	0.748	0.667	0.964	0.737	<0.001
ClusterShadeKep	0.557	0.980	0.558	0.767	0.685	0.967	0.863	<0.001

Note: AUC, the area under curve.

**TABLE 5:** ROC curve analysis of radiomics parameters for differentiating CD4 high expression from low expression in AGC.

Parameter	Cut-off	Sensitivity	Specificity	Accuracy	PPV	NPV	AUC	P
UniformityKtrans	0.498	0.863	0.635	0.748	0.698	0.825	0.778	<0.001
InertiaKep	0.575	0.902	0.673	0.786	0.730	0.875	0.817	<0.001
CorrelationKep	0.691	0.941	0.731	0.835	0.774	0.927	0.856	<0.001

Note: AUC, the area under the curve.

Among the above DCE-MRI radiomics parameters and the clinicopathological data included in this study, only Ktrans (Skewness) was positively correlated with CEA level (*r* = 0.197, *p* = 0.046, Table 6).

**TABLE 6:** Correlation between DCE-MRI parameters and CEA level of AGC.

Kinetic parameter	Parameter	*r* value	*p*-value
Ktrans	Skewness	0.197	0.046*
	Uniformity	−0.149	0.132
Kep	Correlation	−0.039	0.697
	Skewness	−0.007	0.945
	ClusterShade	−0.003	0.972
	Inertia	0.090	0.368

Note: *r*, correlation coefficient. * indicates significance at *p* < 0.05. CEA, carcinoembryonic antigen.

## Discussion

To our knowledge, this is the first study to distinguish the degree of CD4 and CD8 tumor-infiltrating lymphocyte infiltration in AGC patients based on DCE-MRI radiomics characteristics. It has been found that radiomics parameters based on DCE-MRI perfusion maps have good diagnostic performance in distinguishing the degree of CD4 and CD8 infiltration. It is inferred that this approach may help guide the clinical identification of immune therapy beneficiaries and the selection of appropriate treatment options for patients with AGC.

Various biomarkers are currently employed in routine clinical practice, as they can reveal tumor characteristics in a variety of ways [19]. Imaging is a critical examination method for determining the occurrence and progression of tumors. The development of computer algorithms and other technologies has led to the emergence of radiomics. This approach enables the quantification of the pixel and spatial distribution of medical images, and extracted features describe not only the gray level, and texture, but also other information [20]. Most studies have shown that it can reflect tumor heterogeneity and thus be used to evaluate biomarkers noninvasively [21–23]. In this study, we used DCE-MRI to extract radiomics features. Different from traditional MRI imaging techniques, DCE-MRI has the advantage of assessing the tumor vascular microenvironment. DCE-MRI has been applied to several tumor studies and achieved satisfactory results [15]. Therefore, we established DCE-MRI-based radiomics features to identify CD4 and CD8 expression and achieved satisfactory results.

With a high incidence and poor prognosis, gastric cancer is a malignant tumor developing from the stomach mucosal epithelium [24]. In recent years, the host tumor’s local immune response has gradually become a hot research topic [25–27]. Tumor-infiltrating immune cells play a crucial role in this response, and TILs make up the majority of this immune cell [27, 28]. TILs, tumor-infiltrating lymphocytes, are a crucial part of the tumor microenvironment and lymphocytes that leave the bloodstream to infiltrate the tumor. TILs are often linked to a higher immunotherapy response and prognosis, according to a number of studies [29–31]. The number of TILs may be a significant predictor of the response to cytotoxic treatments such as chemotherapy and radiotherapy and is assumed to be associated with the mechanisms regulating cancer growth, progression, and metastasis [32–34]. TILs have major subtypes, CD4^+^ and CD8^+^, which have a significant impact on the prognosis of AGC [35]. Understanding the expression and differences in CD4^+^ and CD8^+^ TILs in patients with advanced gastric cancer can help identify patients, provide reasonable and personalized treatment, and contribute to the advancement of precision medicine. Hyein Ahn et al. discovered that texture features extracted from FDG PET/CT are correlated with CD8^+^ T lymphocytes, which can provide histopathological characteristics of the immune microenvironment and predict cancer patients’ recurrence-free survival [36]. Bian, Y et al. used contrast-enhanced CT to predict tumor CD8^+^ TIL levels and developed an XGBoot classifier with AUCs of 0.75 and 0.67 in the training and validation sets, respectively [37]. Compared to CT and PET/CT, MRI has better resolution and contrast in soft tissue imaging without the need for radiotracers. In this study, MRI-based methods were used, which reflect more functional information about the tumor.

DCE-MRI is an oncology functional imaging technique that measures tumor blood flow, vascular permeability, and vascular and interstitial volume [38]. DCE-MRI has some utility in predicting the expression of various proteins in gastric cancer and assessing gastric cancer angiogenesis [15]. Traditional DCE-MRI parameters are inadequate for assessing intratumoral heterogeneity. Instead of conventional DCE-MRI parameters, a histogram analysis method based on DCE-MRI was used in this study to obtain radiomics parameters. Histogram analysis is a technique for analyzing the gray level distribution on biomedical images and producing metrics that reflect the frequency with which pixels display gray level in a given interval [39, 40]. As a result, radiomics parameters based on histograms are more likely to reflect tumor heterogeneity. DCE-MRI has previously been shown to predict the expression of tumor-infiltrating lymphocytes in breast cancer, lung cancer, and melanoma [41, 42]. According to the findings, radiomics features extracted from DCE-MRI have some utility in identifying the expression of tumor-infiltrating lymphocytes. The feasibility of using DCE-MRI to predict the expression of tumor-infiltrating lymphocyte subsets in AGC patients has not been investigated.

In our study, some pharmacokinetic radiomics parameters based on perfusion maps were found to have good diagnostic performance in distinguishing the different densities of CD4^+^ and CD8^+^ TILs in AGC patients. In this study, ROC curve analysis revealed that ClusterShade of Kep (0.557), skewness of Kep (0.518), and skewness of Ktrans (0.360) provided the perfect combination of sensitivity (0.980, 0.980, and 0.725), specificity (0.558, 0.519, and 0.615), PPV (0.685, 0.667, and 0.649), and NPV (0.967, 0.964, and 0.696) for distinguishing the different densities of CD8^+^ TILs in AGC (*p* < 0.001, respectively). We found that ClusterShade of Kep exhibited the most negative association with CD8^+^ TILs (*r* = −0.630). ClusterShade is one of the texture features of the gray level co-occurrence matrix (GLCM), which is used to describe the distribution of cluster shadows in images. In medical image analysis, ClusterShade is frequently used to analyze heterogeneity and changes in tissue structure, reflecting the distribution of different types of cells within the tissue [39]. The larger the cluster shadow value of Kep indicates the larger the difference in the distribution of different types of cells and the higher the heterogeneity within the tissue [15]. The relationship with CD8^+^ TILs may indicate cell density and arrangement in tumor tissue, thereby having an impact on immune cell infiltration and anti-tumor immune responses. Some studies have shown that tumor cells line more loosely and irregularly tumor tissue, are more easily attacked by immune cells, and achieve better immunotherapeutic responses [29]. Therefore, Kep.ClusterShade may be a potential biomarker to predict response to immunotherapy.

As for CD4, the ideal combination of sensitivity (0.941, 0.902, and 0.863), specificity (0.731, 0.673, and 0.635), PPV (0.774, 0.730, and 0.698), and NPV (0.927, 0.875, and 0.825) was Correlation of Kep (0.557), Inertia of Kep (0.518), and Uniformity of Ktrans (0.360). (*p* < 0.001), respectively. The negative connection between Kep and CD4^+^ TILs was the strongest (*r* = −0.616). Kep represents the transfer rate constant between plasma and extracellular space, and it is a reflection of extravascular interstitial permeability. Previous studies have suggested that Kep may reflect microvessel density characteristics such as vessel area [43]. A higher K_ep_ value represents an increase in tumor perfusion and vascular permeability, which is attributed to an increased number of nascent tumor vessels [44]. CD4^+^ T cells play an important role in the tumor microenvironment, and there is no direct evidence that CD4^+^ T cells directly inhibit tumor angiogenesis. However, it has been shown that CD4^+^ T cells can also secrete some cytokines, such as IL-10, which can inhibit tumor angiogenesis through a variety of pathways, such as inhibiting endothelial cell proliferation and migration, and reducing the secretion of vascular endothelial growth factor [9]. Lower CD4 numbers may indirectly lead to more aggressive tumors and more angiogenesis, thus resulting in a negative correlation between Kep and CD4. The mechanism still needs further study in the future. Previous studies have shown that an insufficient number of CD8^+^ T lymphocytes can cause tumor cell proliferation, invasion, and metastasis, resulting in rapid tumor progression [45–47]. Numerous studies have demonstrated that a positive prognosis for gastric cancer is related to high expression of TILs (CD4, CD8) [35, 48]We find that the derived parameters of gray level co-occurrence matrix (GLCM) play an important role. GLCM played an important role in predicting CD8^+^ TIL density, which is consistent with previous reports that radiomics metrics of GLCM were dominant in models predicting CD8^+^ TIL density in renal clear cells [49]. This study also found a close relationship between the radiomics parameters of GLCM and CD4^+^TILs.

In addition, a correlation was observed between Ktrans (skewness) and CEA levels (*r* = 0.197, *p* = 0.046) in our study. Carcinoembryonic antigen (CEA) is a widely used tumor marker in patients with gastrointestinal cancer before treatment and during follow-up. Previous studies have found that patients with high pretreatment serum CEA levels have a poor prognosis [50]. Currently, the mechanism by which elevated CEA levels lead to worse survival remains unclear. One possible explanation is that CEA acts indirectly in cell-cell adhesion between tumor cells and vascular endothelial cells, thereby contributing to tumor invasion and metastasis [51]. Patients with higher CEA levels in this study had higher Ktrans (skewness) values relative to patients with normal CEA levels. Higher Ktrans (skewness) values in tumor tissue represent a more heterogeneous distribution of tumor vessels, that is, more heterogeneous vascularity [15]. The association between them may therefore be plausible, however, to better elucidate this association, we should investigate this association further.

This study has certain limitations. Firstly, the TIL subtypes investigated in this study included only CD4 and CD8, and other subtypes were not included. Future studies should include other subtypes associated with immunotherapy to more comprehensively assess the tumor immune microenvironment. Secondly, the study did not include other molecular expression data, such as EBV or MSI status. The inclusion of these data allows better patient stratification and may provide a basis for precision immunotherapy. In future investigations, we plan to include these additional molecular expression data to further enrich the study and strengthen its clinical relevance. Thirdly, it is important to highlight that the current study lacks clinical validation. While our findings provide valuable insights, further studies with larger sample sizes and prospective designs are needed to validate the results in a clinical setting. Clinical validation would help assess the practical utility and generalizability of our findings, ensuring their applicability in real-world clinical practice. Last but not least, this study is a single-center retrospective study with possible selection bias, and more patients (based on existing studies) will be included in the future for further multicenter studies to verify. These limitations should be considered when interpreting the results of our study and provide directions for future research to overcome these limitations and further advance the field. In conclusion, our study showed that some quantitative pharmacokinetic parameters obtained from DCE-MRI have the potential to assess the levels of CD8^+^ and CD4^+^ TILs infiltration. The identification of CD8^+^ and CD4^+^ TIL infiltration levels is helpful for the development of precision medicine and provides more information for clinical practice to assist clinical decision-making. In the future, machine learning methods will be introduced to establish better evaluation models.

## Conclusion

In several cases, the expression of CD4^+^ and CD8^+^ TILs in AGC patients correlated with DCE-MRI quantitative perfusion histogram characteristics. Quantitative perfusion histogram parameters using DCE-MRI can be utilized as imaging biomarkers to noninvasively reflect CD4^+^ and CD8^+^ TIL expression in AGC patients and serve as a reference for customized treatment of AGC patients.

## Data Availability

The raw data supporting the conclusion of this article will be made available by the authors, without undue reservation.
